# Genomic And Tumor Microenvironment Differences Between Cell Cycle Progression Pathway Altered/Non-Altered Patients With Lung Adenocarcinoma

**DOI:** 10.3389/fonc.2022.843528

**Published:** 2022-02-28

**Authors:** Guangyao Shan, Guoshu Bi, Yunyi Bian, Besskaya Valeria, Dejun Zeng, Huan Zhang, Guangyu Yao, Yi Zhang, Hong Fan, Cheng Zhan

**Affiliations:** ^1^ Department of Thoracic Surgery, Zhongshan Hospital, Fudan University, Shanghai, China; ^2^ Department of Thoracic Surgery, Zhongshan Hospital, Fudan University (Xiamen Branch), Xiamen, China

**Keywords:** cell cycle, lung adenocarcinoma, medical informatics, RNA-seq, tumor microenvironment

## Abstract

**Background:**

Identified as a hallmark of cancer, the dysregulated cell cycle progression plays an important role in the promotion and progression of lung adenocarcinoma (LUAD). However, the genomic and microenvironment differences between cell cycle progression pathway altered/non-altered LUAD patients remain to be elucidated.

**Materials and Methods:**

Data of this study were obtained from The Cancer Genome Atlas (TCGA), including simple nucleotide variation, copy number variation (CNV), RNA-seq gene expression, miRNA expression, survival, and clinical information. Besides, 34 LUAD samples from our institution were used as a validation cohort. Differentially expressed genes (DEGs), enrichment analysis, and immune cell infiltration were detected. At last, we built a LASSO-binary Logistic regression model to predict the cell-cycle-related gene mutation (CDKN2A, CCND1, CDK4, CCNE1, and RB1) in LUAD patients and further verified it in the samples from our institution.

**Results:**

Based on the cell cycle progression pathway status, the LUAD patients were divided into the mutation (n=322) and wild (n=46) groups. Compared to the wild group, the mutation group had a higher mutational load and CNV. Among the 16684 protein-coding genes analyzed, 302 were upregulated, and 354 were downregulated in the mutation group. Enrichment analysis indicated that these DEGs were closely related to metabolism items. After performing immune cell infiltration analysis of 22 immune cells, we found the proportion of 5 immune cells such as monocytes (P<0.01) and dendritic cells (P<0.01) were higher in the wild group. Finally, a cell-cycle-related 15-signature model was built by LASSO-Logistic regression analysis, which could predict the cell cycle progression pathway-related gene mutation (CDKN2A, CCND1, CDK4, CCNE1, and RB1) in LUAD patients. The validation cohorts showed the sensitivity and specificity of this model were 0.667 and 0.929, respectively.

**Conclusion:**

The genomic and microenvironment characteristics differed between the cell cycle progression pathway altered/non-altered patients with LUAD. Our findings may provide new insight into personalized treatment for LUAD patients.

## Introduction

Lung cancer is the leading cause of cancer-related death and the most common type of cancer in men globally (1). The two major types of lung cancer are non-small-cell lung cancer (NSCLC) and small-cell lung cancer. The former type accounts for 85% of lung cancer cases (2), among which lung adenocarcinoma (LUAD) is the most common subtype with more than 500,000 deaths each year worldwide (3). According to the report published by the American Cancer Society and the National Cancer Institute, the 5-year relative survival of NSCLC for all stages is only 23% (4). In the past decades, the clinical application of targeted therapies and immunotherapies alone or in combination with conventional therapies such as chemotherapies has greatly improved the prognosis of LUAD patients (5), and they begin to play an increasingly important role in the treatment of LUAD. These remarkable achievements are attributed to the deep understanding of the molecular traits of LUAD.

The cell cycle, a highly organized process regulated by cyclin and cyclin-dependent kinase (CDK) complexes and other regulators, consists of four sequential phases: G1, S, G2, and M phases (6). In the G1 phase, cells are busy with biosynthesis prepared for the following steps. S phase is characterized by DNA replication and the synthesis of related proteins like histones. During the G2 phase, cells enhance the lipid synthesis required for membrane construction and guarantee that everything is ready to initiate the mitosis (6). In the M phase, the chromosomes will be evenly separated into two daughter cells, and then the mitosis comes to an end. Compared to normal tissues that could carefully control the production and release of growth-promoting signals during the cell cycle, cancer cells could maintain sustainable proliferative signals and avoid programs that may arrest cell cycle progression (7). We found the five genes, including cyclin-dependent kinase inhibitor 2A (CDKN2A), cyclinD1 (CCND1), cyclin-dependent kinase 4 (CDK4), cyclinE1 (CCNE1), and retinoblastoma 1 (RB1), are frequently aberrant in LUAD patients, and they orchestrate a pathway together to regulate cell cycle progression (8). CDKN2A, which encodes the CDK inhibitor p16^INK4a^ and p14^ARF^ protein, is located upstream of the cell cycle progression pathway and could be found loss-of-function in multiple human tumors, including NSCLC (9). As a tumor suppressor gene, CDKN2A functions as an inhibitor of CDK4, which plays an important role in the G1 phase. CDK4-CCND1 complexes, along with CDK2-CCNE1 complexes, could phosphorylate and inactivate the RB1 to release the E2F transcription factor, thus inducing the cell to complete G1-to-S transition (8, 10, 11). Among all the mitotic steps mentioned above, the transition from G1 to S is the most crucial for cell cycle progression. Once the cell has entered the S phase, it is bound to get through S, G2, and M phases and generate two daughter cells (12). As the cell cycle progression pathway is well organized by various regulators, errors at any step may disrupt the homeostasis of cell proliferation and death, thus contributing to tumor promotion and progression.

In this article, we systematically analyzed the differences in somatic mutations, genomic expression, and immune cell infiltration between the cell cycle progression pathway altered and non-altered patients with LUAD. This study aims to enhance our understanding of the function of the cell cycle progression, which could shed new light on the development of new drugs targeting this pathway.

## Materials and Methods

### Data Collection and Processing

The data of this study, including simple nucleotide variation, copy number variation (CNV), RNA-seq gene expression, miRNA expression, survival, and clinical information, were retrieved from The Cancer Genome Atlas (TCGA) data portal (https://portal.gdc.cancer.gov/). 368 patients with intact information of simple nucleotide variation, CNV, RNA-seq gene expression, miRNA expression, survival, and clinical features were included in this study for further analysis in R software (version 4.0.3).

We also collected 34 LUAD samples from patients who underwent lobectomy and systematic lymph node resection at the Department of Thoracic Surgery, Zhongshan Hospital, Fudan University between 2016 and 2017. All pulmonary resections were performed by professional thoracic surgeons in our institution, and the diagnoses of LUAD were confirmed by at least two qualified pathologists. All patients have signed informed consent to conduct genomic studies consistent with the ethical principles of the Declaration of Helsinki. Patients with a history of chemotherapy, radiotherapy, and immunotherapy or who had evidence of metastasis were excluded. RNA sequencing for all tumor samples was performed using Illumina Hiseq 2500 and BGI-500RNAseq platforms *(* **Supplementary Material 1** *)*. Besides, CDKN2A, CCND1, CDK4, CCNE1, and RB1 mutations in our samples were identified by whole exon sequencing. GATK4 software was used to detect single nucleotide polymorphisms (SNPs), insertions, and deletions (Indels). Only SNPs and Indels with quality/depth ratio >=2.0 were considered for subsequent analysis and were annotated using Annovar software. The study was approved by the ethical committees of Zhongshan Hospital (No. 201986122).

### Clinical Features of the Patients and Survival Analysis

Patients who harbored any of the designated cell cycle progression pathway-related gene mutation (CDKN2A, CCND1, CDK4, CCNE1, and RB1) were defined as the mutation group, and the remaining were classified as the wild group. Clinical characteristics including age, gender, race, anatomic location of the lesion, smoking history, and TNM stage were compared between the two groups. Next, Kaplan-Meier survival analysis was performed, and log-rank test was used to show the difference between the two groups.

### Differentially Mutated Genes (DMGs)

The simple nucleotide variation data were stratified into two groups, and their mutational patterns were investigated separately. DMGs were detected using Fisher exact test and visualized by *maftools* package. Besides, mutational load and CNV were calculated for every patient, and their differences between the two groups were explored.

### Differentially Expressed Genes (DEGs) and Enrichment Analysis

After removing the genes whose average expression levels in all patients were less than 10, we used *edgeR* package to explore the protein-coding DEGs based on the RNA-seq gene expression data (count format). The absolute value of Log_2_FoldChange (|Log FC |) >1 and p-value <0.05 were considered as significantly different.

Gene Ontology (GO) and Kyoto Encyclopedia of Genes and Genomes (KEGG) enrichment analysis were performed based on the DEGs using *org.Hs.eg.db* and *Clusterprofiler* package. Subsequently, we performed gene set enrichment analysis (GSEA) based on the 16684 protein-coding genes. Adjusted p-value less than 0.05 was considered as significantly different. Finally, we used gene set variation analysis (GSVA) to investigate the difference of cell-cycle-related events *(* **Supplementary material 2** *)* between the two groups. Rather than ssGSEA, we harnessed the GSVA method in this study because GSVA includes the normalization of gene expression to reduce the noise of the data and has been shown to outperform ssGSEA when measuring the signal-to-noise ratio in differential gene expression and differential pathway activity identification analyses (13).

### Protein-to-Protein Interaction (PPI) Network and Cluster Analysis

To further investigate the inner correlation of the DEGs, we mapped the DEGs in STRING (14) (https://cn.string-db.org, version 11.0b) to build a PPI network. Interaction with a confidence score greater than 0.4 was included in the network. Subsequently, the Molecular Complex Detection (MCODE) plugin in Cytoscape (https://cytoscape.org, version 3.8.2) was harnessed to identify modules of the PPI network (degree cut-off=2, node score cut-off=0.2, k-score=2, and max depth=100).

### Competing Endogenous RNA (ceRNA) Network

Differentially expressed miRNAs were detected by *limma* package using the miRNA expression data (TPM normalized). The criteria for defining differentially expressed miRNAs were set as follows: |Log FC| >0.5 and p-value <0.05. Next, we investigated differentially expressed long non-coding RNAs (lncRNAs) between the two groups, and the thresholds for |Log FC| and p-value were the same as the protein-coding DEGs.

After matching the differentially expressed miRNAs with DEGs and differentially expressed lncRNAs using miRWalk (http://mirwalk.umm.uni-heidelberg.de) and miRcode (http://www.mircode.org) webtools, a ceRNA network of DEGs - differentially expressed miRNAs -differentially expressed lncRNAs was constructed in Cytoscope.

### Characteristics of Immune Cell Infiltration

The profiling of 22 immune cell types was analyzed using RNA-seq gene expression data (FPKM format) and the leucocyte signature matrix (LM22) in CIBERSORT (https://cibersort.stanford.edu). LM22, composed of 547 genes, could distinguish 22 human hematopoietic cells, including various subtypes of T cells, B cells, plasma cells, NK cells, and myeloid cells (15). Subsequently, we compared the immune and stromal scores between the two groups by *estimate* package. At last, we detected the expression levels of 15 immune checkpoints and 20 co-stimulators between the two groups. The relationships between these immune molecules were detected by Spearman correlation analysis and visualized by *ggcorrplot* package.

### Construction of the Predictive Model

After taking the intersection of the DEGs and 1872 cell-cycle-related genes *(* **Supplementary material 3** *)*, we obtained 34 cell-cycle-related DEGs (ccDEGs). Based on the ccDEGs, we performed least absolute shrinkage and selection operator (LASSO) and subsequent binary logistic regression analysis to build a model which could predict if a LUAD patient harbored specific cell cycle progression pathway-related gene (CDKN2A, CCND1, CDK4, CCNE1, and RB1) mutation. The LASSO regression analysis is a penalized method to select data with high dimensions and reduce the impact of overfitting (16, 17). Ten-fold cross-validation was adopted using the *glmnet* package to determine the optimal parameter λ and corresponding genes. The cut-off value of this model was determined by the receiver operating characteristic curve (ROC). Next, we explored the effect of the candidate genes on proliferation. The CERES dependency scores of these candidate genes in 51 LUAD cell lines were retrieved from the Cancer Dependency Map (DEPMAP, https://depmap.org/portal/), which represented the effect on cell viability after knocking out corresponding genes by CRISPR-Cas9 genetic perturbation reagents (18). A score of zero indicated that a gene was not essential. Scores less than zero indicated knockout of the corresponding genes could inhibit cell proliferation; the smaller the score, the more pronounced the effect. Scores greater than zero showed the opposite effect.

### Statistical Analysis

All statistical analyses were conducted in R software (version 4.0.3). The comparison of the clinical features of the LUAD patients between the two groups was carried out, of which categorical variables were compared by Chi-square test or Fisher exact test when appropriate and continuous variables were compared by Student’s t-test. The Student’s t-test was also used to compare continuous variables such as the mutational load, immune and stromal scores. Log-rank test was used to compare overall survival between the two groups in Kaplan-Meier survival analysis. All the tests used in our study were two-sided, and the significance threshold of p-value was set as 0.05.

## Results

### Clinical Features of the Patients and Survival Analysis

**Figure 1:**
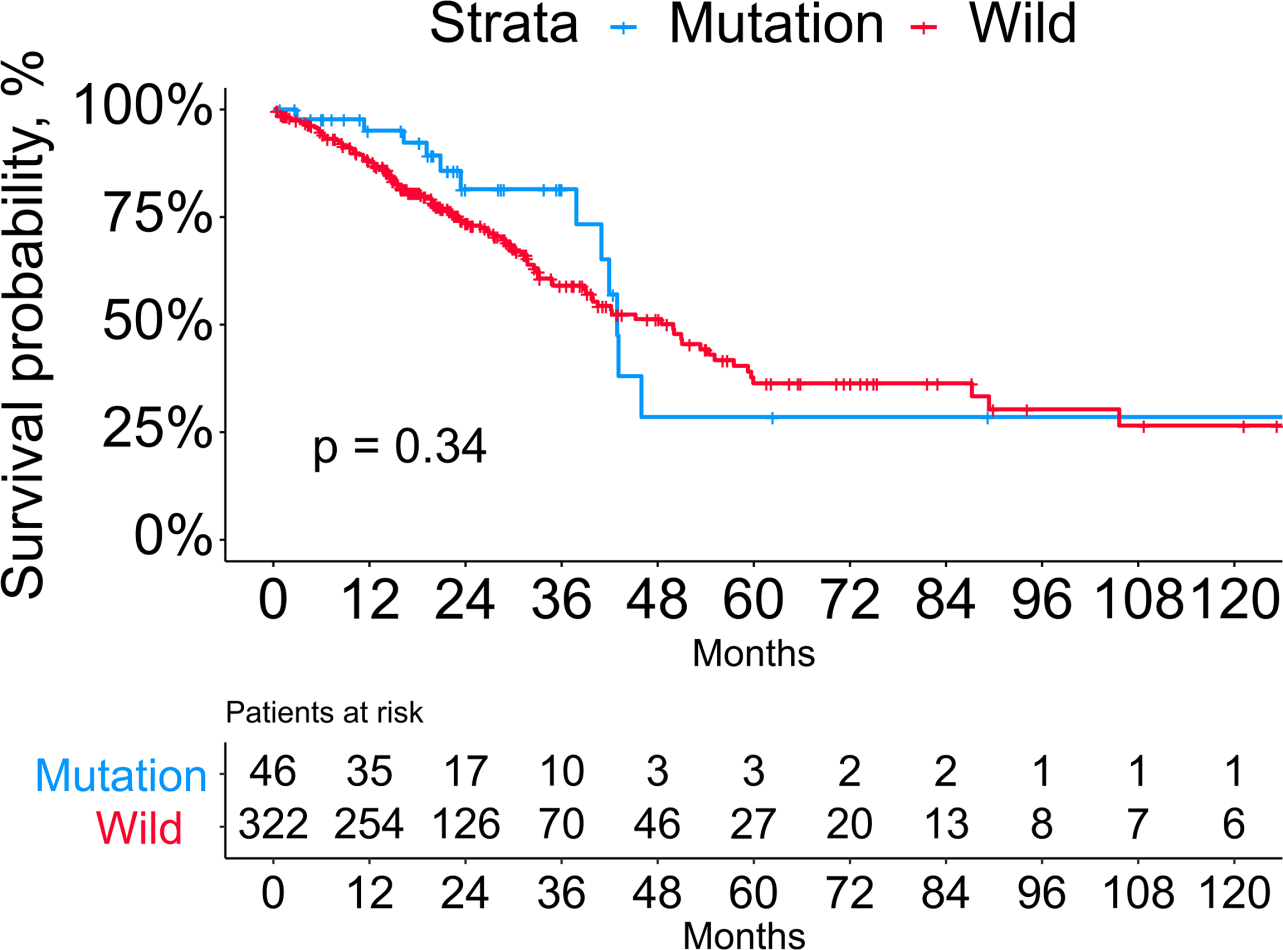
Kaplan-Meier survival analysis between the mutation and wild groups.

The mutational rates of RB1, CDKN2A, CCNE1, CDK4, and CCND1 in all LUAD patients included in our study were 8.2%, 3.5%, 1.4%, 0.5%, and 0.3%, respectively. After dividing the patients into mutation (n=46) and wild (n=322) groups, we found that more than half of the patients in the mutation group harbored RB1 mutation (65.2%, the common variants in lung cancer were C706F, G748K, and R661W), followed by CDKN2A (28.3%, the common variants in LUAD were R80Q and H83Y), CCNE1 (10.9%), CDK4 (4.3%, the common variants in LUAD were R24L and R24C) and CCND1 (2.2%). Besides, 5 of 46 patients in the mutation group harbored more than one mutant gene mentioned above. Comparison of the baseline information showed no significant differences between the two groups in age, gender, anatomical location of the lesion, smoking history, and TNM stage (**Table 1**). Next, we performed the Kaplan-Meier survival analysis between the two groups, but log-rank test showed no significance (**Figure 1**). As the number of patients in the mutation group was much smaller than the wild group, this result needs to be further verified in larger cohorts.

**Table 1:** Clinical features of the LUAD patients.

	Mutation *(N = 46)*	Wild *(N = 322)*	P-value
**Race**			*0.019*
Black	10 (21.7%)	36 (11.2%)	
White	34 (73.9%)	283 (87.9%)	
Yellow	2 (4.4%)	3 (0.9%)	
**Age**			*0.301*
Mean [Q1, Q3]	62.5 [57.2;72.0]	66.0 [59.0;73.0]	
**Gender**			*1.000*
Female	25 (54.3%)	178 (55.3%)	
Male	21 (45.7%)	144 (44.7%)	
**Smoking history**			*0.291*
Current smokers	15 (32.6%)	76 (23.6%)	
Never smokers	4 (8.7%)	48 (14.9%)	
Previous smokers	27 (58.7%)	198 (61.5%)	
**Stage**			*0.851*
Stage I	26 (56.5%)	181 (56.2%)	
Stage II	11 (23.9%)	76 (23.6%)	
Stage III	6 (13.0%)	51 (15.8%)	
Stage IV	3 (6.5%)	14 (4.4%)	
**Anatomic location of the lesion**			*0.161*
Lower lobe, lung	15 (32.6%)	106 (32.9%)	
Lung	0 (0.0%)	6 (1.9%)	
Main bronchus	1 (2.2%)	0 (0.0%)	
Middle lobe, lung	0 (0.0%)	18 (5.6%)	
Overlapping lesion of lung	0 (0.0%)	2 (0.6%)	
Upper lobe, lung	30 (65.2%)	190 (59.0%)	

### Mutational Events

**Figure 2:**
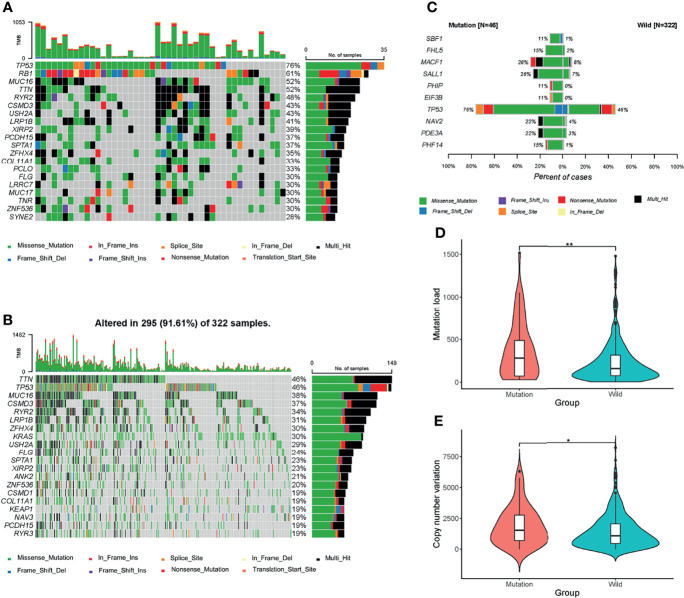
Mutational patterns of the two groups. **(A, B)** Waterfall maps of the top 20 mutant genes in the mutation and wild groups. **(C)** Top 10 most significant differentially muted genes (DMGs) between the mutation and wild groups. Genes including CDKN2A, CCND1, CCNE1, CDK4, and RB1 were excluded. **(D, E)** Mutation load and copy number variation (CNV) between the two groups. *P < 0.05; **P < 0.01.

Different mutational patterns were detected between the mutation and wild groups (**Figure S1**). The top 20 mutant genes in the mutation and wild groups were shown separately (**Figures 2A, B**), among which fourteen ones were shared by the two groups, and all of them were observed with higher mutational rates in the mutation group. Next, we used Fisher exact test to detect the DMGs between the two groups. 286 genes were defined as DMGs (**Figure 2C**, **Supplementary material 4**), 30 of which such as PDE3A (22% mutation group *vs.* 3% wild group, *P<0.0001*) and TP53 (76% mutation group *vs.* 46% wild group, *P<0.0001*) were related to cell cycle events.

At last, we compared the mutational load and CNV between the two groups. The mutation group had a higher level of mutational load (**Figure 2D**) and CNV (**Figure 2E**), which indicated that the mutation group harbored more mutational events and thus is more likely to benefit from immunotherapies.

### DEGs and Enrichment Analysis

**Figure 3:**
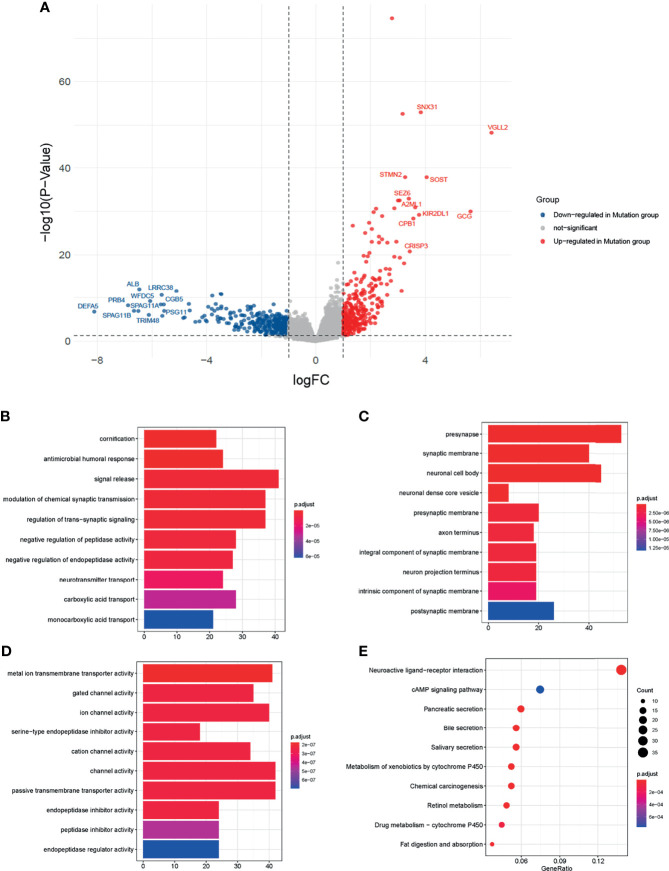
Transcriptomics analysis of the two groups. **(A)** Volcano map of differentially expressed genes (DEGs) between the mutation and wild groups. Blue dots represented genes downregulated in the mutation group. Grey dots represent genes not significant between the two groups. Red dots represented genes upregulated in the mutation group. **(B–D)** Barplots of the top ten significantly different biological processes (BP), cellular component (CC), and molecular function (MF) by Gene Ontology (GO) enrichment analysis. **(E)** Dot plot of the top ten significantly different enrichment pathways by Kyoto Encyclopedia of Genes and Genomes (KEGG) analysis.

Among the 16684 protein-coding genes analyzed, 302 were upregulated and 354 were downregulated in the mutation group (**Figure 3A**, **Supplementary Material 5)**. Among the DEGs, VGLL2 (logFC=6.41, *P<0.0001*), GCG (logFC=5.64, *P<0.0001*), and SOST (logFC=4.04, *P<0.0001*) were the most significantly upregulated genes; DEFA5 (logFC= -8.11, *P<0.0001*), PRB4 (logFC= -6.87, *P<0.0001*), and SPAG11B (logFC= -6.66, *P<0.0001*) were the most significantly downregulated genes.

To explore the biological functions affected by DEGs, we performed the GO and KEGG analysis. In GO analysis (**Figures 3B–D**), the top 3 of the 630 significantly different biological processes were cornification, antimicrobial humoral response, and signal release. KEGG analysis showed the DEGs were related to fat digestion and absorption, drug metabolism − cytochrome P450, and retinol metabolism (**Figure 3E**).

**Figure 4:**
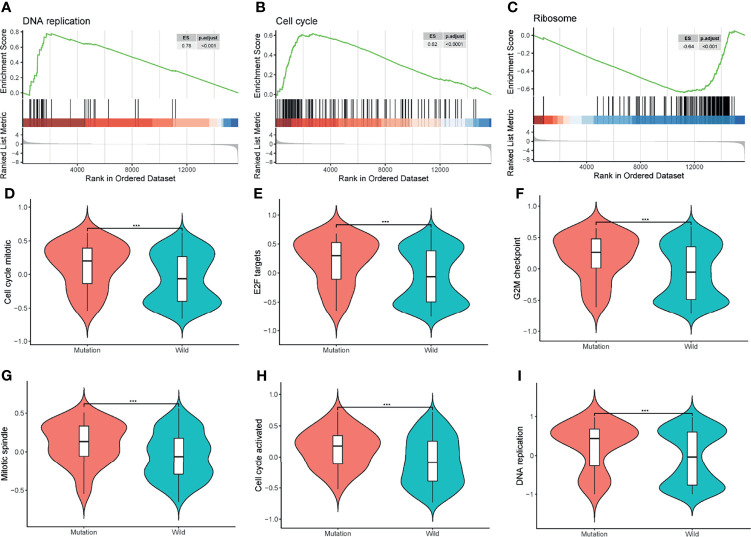
Gene Set Enrichment Analysis (GSEA) and Gene Set Variation Analysis (GSVA). **(A–C)** GSEA of all the protein-coding genes between the two groups. ES, enrichment score; p.adjust, adjusted p-value. **(D–I)** GSVA of the cell cycle-related events between the mutation and wild groups. ***P < 0.001.

Apart from GO and KEGG analysis, we further investigated the influence of cell cycle progression pathway status using GSEA and GSVA analysis. Processes like cell cycle, DNA replication, and ribosome were significantly different between the two groups by GSEA analysis (**Figures 4A–C**). GSVA analysis showed cell-cycle-related events such as DNA replication, E2F targets, mitotic spindle, and G2M checkpoints were significantly different between the two groups (**Figures 4D–I**).

### PPI Network and Cluster Analysis

**Figure 5:**
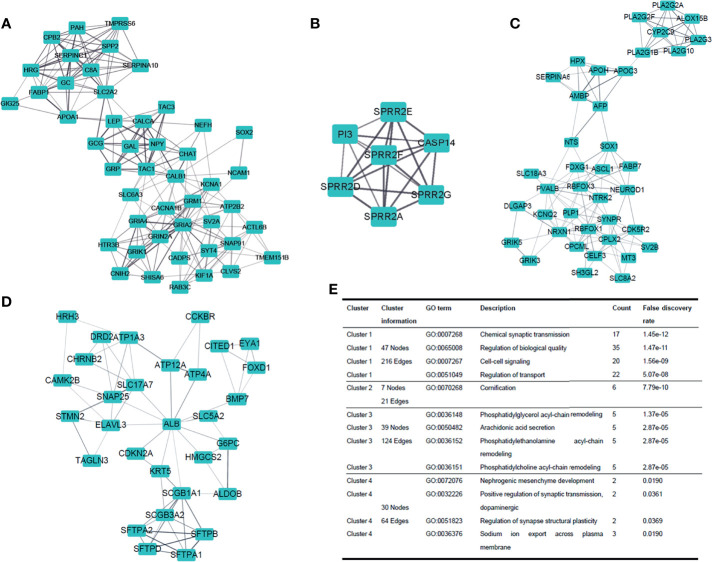
Dissection of protein-to-protein interaction. **(A–D)** Top four clusters in the protein-to-protein interaction (PPI) network. Line thickness indicates the strength of data support. **(E)** GO enrichment analysis of the four clusters, respectively.

PPI clusters were investigated by MCODE (19) in Cytoscape. The top four modules (clustering scores were 9.391, 7.000, 6.526, and 4.414, respectively) are shown in **Figures 5A–D**. Next, we carried out the GO enrichment analysis of the four clusters separately. The result demonstrated that these clusters were related to biological processes such as cell-cell signaling, cornification, arachidonic acid secretion, and nephrogenic mesenchyme development (**Figure 5E**).

### ceRNA Network

Both miRNAs and lncRNAs could act as important modulators which may influence the expression and function of mRNAs. Here, we analyzed the differentially expressed miRNAs and lncRNAs between the two groups (**Figure S2**, **Supplementary material 5)**. The results showed that 31 miRNAs were upregulated in the mutation group, the most significantly different ones of which were miR−3689e (logFC=2.30, *P<0.01*), miR−3689f (logFC=1.95, *P<0.01*), and miR−1197 (logFC=1.70, *P<0.001*). 5 miRNAs were downregulated in the mutation group, and the most significantly different one was miR−203b−3p (logFC=-0.86, *P<0.001*). For lncRNAs, LINC01305 (logFC=3.21, *P<0.0001*) was the most significantly different one among 31 upregulated lncRNAs in the mutation group; AF003626.1 (logFC=-4.63, *P<0.0001*) was the most significantly different one among 64 downregulated lncRNAs.

**Figure 6:**
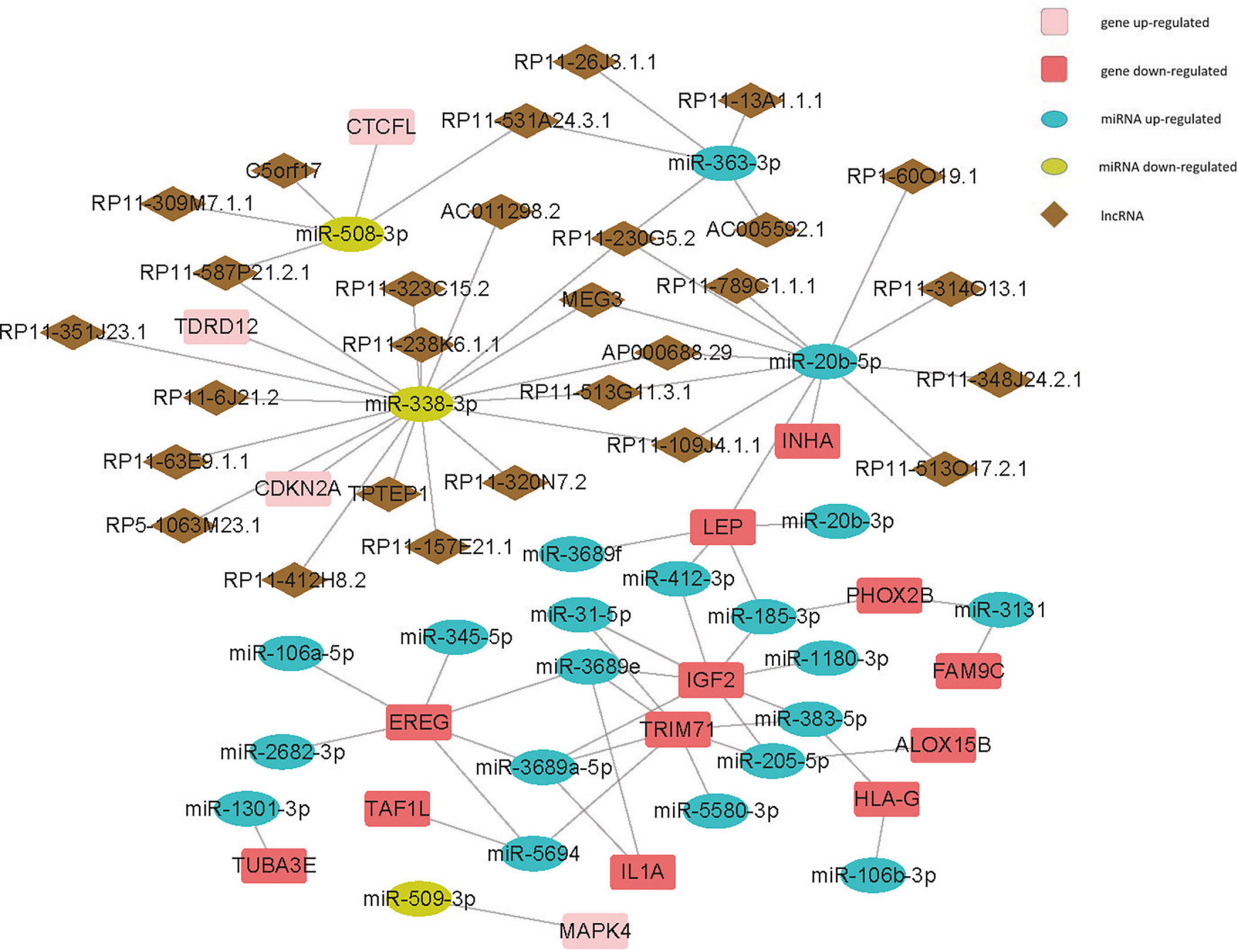
Competing endogenous RNA (ceRNA) network of cell-cycle-relatedDEGs - differentially expressed miRNAs - differentially expressed lncRNAs.

After matching DEGs and differentially expressed lncRNAs with differentially expressed miRNAs, we built a ceRNA network (**Figure 6**, **S3**, **Supplementary material 6**) to elucidate their relationships (Here, only genes negatively regulated by miRNAs were shown in this network). We found that both miR-3131 and miR-185-3p could regulate more than 40 genes, suggesting the two miRNAs and the genes they regulate might play a significant role in the differences between the mutation and wild group.

### Characteristics of Immune Cell Infiltration

**Figure 7:**
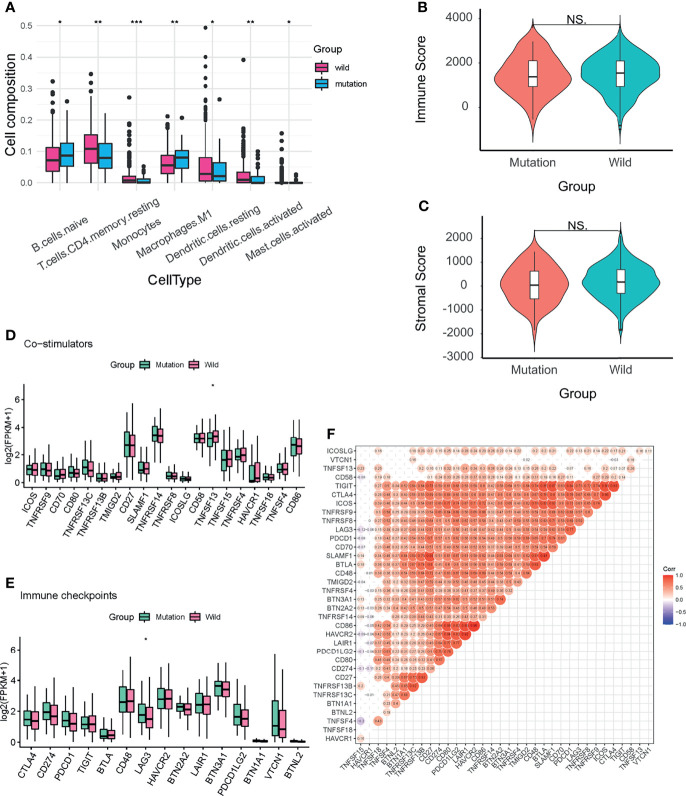
Immune infiltration analysis of the two groups. **(A)** Seven immune cell types with significant differences between the two groups. **(B, C)** Stromal and immune scores of the two groups. **(D, E)** Expression levels of fifteen immune checkpoints and twenty co-stimulators between the two groups. **(F)** Spearman correlation analysis of the immune checkpoints and co-stimulators. Labels in the circles indicated the correlation coefficients of the corresponding variables. Correlation coefficients without significant levels were hidden. NS, not significant; *P < 0.05; **P < 0.01; ***P < 0.001.

Tumor immune cell infiltration refers to the migration of immune cells from the peripheral blood to the tumor tissue, where they exert their function (20). Different immune cell infiltration patterns were detected between the two groups (**Figure 7A**, **S4**)using CIBERSORT (15) (permutations set =1000). Among all the significantly different cell types, we found that the proportion of naïve B cells (*P<0.05*) and M1 macrophages (*P<0.01*) were higher in the mutation group, while CD4^+^ memory resting T cells (*P<0.01*), monocytes (*P<0.001*), activated mast cells (*P<0.05*), resting (*P<0.05*) and activated (*P<0.01*) dendritic cells (DCs) were higher in the wild group. Next, we further explored the tumor purity between the mutation and wild groups, while we failed to find any significant difference between the two groups (**Figures 7B, C**).

Then we analyzed the expression levels of 15 immune checkpoints and 20 co-stimulators between the two groups (**Figures 7D, E**). Among co-stimulators, TNFSF13 showed a significant difference (*P<0.05*) and was higher in the wild groups. As for immune checkpoints, LAG3 was significantly higher (*P<0.05*) in the mutation group. The Spearman correlation test demonstrated that most of these immune molecules were positively correlated (**Figure 7F**).

### Construction of the Predictive Model

The optimal parameter λ of LASSO analysis was set as the smallest partial likelihood deviance (**Figure S5A, B**). A panel of fifteen ccDEGs including SOX2, IGF2, CDKN2A, EREG, BMP7, IL1A, HEPACAM2, MAPK4, SOX11, FBXO43, TDRD12, INSC, MOV10L1, PIWIL2, and NLRP5 were employed (**Figure S6**). Subsequently, we conducted the binary Logistic regression analysis to get the coefficients of each item, and the predictive model could be demonstrated as the following formula: score = 0.042*expression level of SOX2+ (-0.2086)*expression level of IGF2+ 0.6522*expression level of CDKN2A+ (-0.1066)*expression level of EREG+ 0.1315*expression level of BMP7+ (-0.3672)*expression level of IL1A+ 0.0585*expression level of HEPACAM2+ 0.1757*expression level of MAPK4+ 0.3392*expression level of SOX11+ 0.7282*expression level of FBXO43+ 0.2677*expression level of TDRD12+ (-1.9734)*expression level of INSC+ 0.1685*expression level of MOV10L1+ 0.7578*expression level of PIWIL2+ (-1.2477)*expression level of NLRP5 (**Figure S5C**). ROC demonstrated the cut-off value of this model was -2.466, and the area under the curve (AUC) is 0.845 (**Figure S5**D). When the score is less than -2.466, the LUAD patient is more likely to harbor CDKN2A/CCND1/CDK4/CCNE1/RB1 mutations. In the validation cohort from our institution, 28 were found to harbor CDKN2A/CCND1/CDK4/CCNE1/RB1 mutations by whole exon sequencing. After testing the model in our samples, we found the sensitivity and specificity of this model were 0.667 and 0.929, separately.

After obtaining the CERES scores of the fifteen ccDEGs *(* **Supplementary material 7** *)*, we found the CERES scores of five ccDEGs (BMP7, IGF2, MAPK4, TDRD12, and NLRP5) were greater than zero in more than 3/4 LUAD cell lines, suggesting they’re more likely to exert an anti-proliferation effect in LUAD patients. On the contrary, four ccDEGs (FBXO43, IL1A, INSC, and SOX2) were thought to promote proliferation, given that their CERES scores were less than zero in more than 3/4 LUAD cell lines.

## Discussion

Lung cancer is the leading cause of cancer-related death worldwide, among which LUAD has been the most prevalent histopathological type since 1985 (21). In our study, we explored the differences between the CDKN2A-CDK/cyclin-RB1 cell cycle progression pathway altered/non-altered patients with LUAD in somatic mutation, genomic expression, and immune cell infiltration. Compared to the wild group, the mutation group had a higher level of mutational load and CNV. By analyzing the RNA-seq gene expression data, we found that 302 protein-coding genes were upregulated in the mutation group, such as VGLL2, and 354, including DEFA5, were downregulated. Through enrichment analysis, the DEGs were found closely related to metabolism items. As for immune cell infiltration, we found that 7 out of 22 cell types were significantly different between the two groups. The proportion of CD4^+^ memory resting T cells (*P<0.01*), monocytes (*P<0.001*), activated mast cells (*P<0.05*), resting (*P<0.05*), and activated (*P<0.01*) dendritic cells were higher in the wild group.

Most RB1-mutant tumors also harbor TP53 co-alterations (22). In our study, thirty patients had RB1 mutation, twenty-two (73.3%) of whom also carried TP53 mutation. As a well-known tumor suppressor gene, TP53 could monitor the abnormalities within the cell. Once the cell suffers excessive DNA damage or the status is not optimal for mitosis, TP53 will suspend the progression of the cell cycle in time until the conditions have been normalized. Besides, when a cell encounters irreversible impairment, TP53 could trigger apoptosis to maintain homeostasis (7). Mutant TP53 not only loses the abilities of surveillance but also creates a circumstance that favors immune evasion and tumor progression by disrupting the innate immune signaling (23). Given the important roles RB1 and TP53 play in the cell cycle progression and the high mutational rates in LUAD patients, both may be potential targets for drug development.

The mutation group had a higher level of mutational load (*P<0.01*), indicating that these patients are likely to produce more neo-antigens that could be recognized by T cells (24). Therefore, the mutation group may acquire a better clinical outcome to immunotherapies than the wild group. Aside from mutational load, LAG3, an immune checkpoint molecule, is also higher in the mutation group. Immune checkpoints are crucial for maintaining autoimmune tolerance but could be harnessed by cancer cells to evade immune surveillance. The advent of anti-PD1 and PD-L1 drugs has shown great success in lung cancer patients. However, less than 25 percent of patients are expected to benefit from them (25). LAG3 could be detected on tumor-infiltrating lymphocytes, B cells, natural killer cells, and Tregs (26). With nearly 20% sequence identical to CD4, it could bind with MHC-II, thus negatively regulating the function of NK cells, CD4^+^ and CD8^+^ T cells, and Tregs (27). In NSCLC patients, LAG3 is usually associated with PD-1 expression and poor prognosis (25). It is reported that LAG3 may have a synergistic effect with PD-1/PD-L1. Therefore, LAG3 may provide new strategies to improve the effects of anti-PD1 and PD-L1 therapies, and patients with altered cell cycle progression pathways are likely to benefit from it.

Compared to the wild group, DEFA5 is significantly downregulated in the mutation group. Defensins are small cysteine-rich cationic polypeptides that are secreted by specific leukocytes and epithelial cells (28). Recognized as antimicrobial agents, defensins also play an important anti-tumor role (29). *Via* binding with BMI1 protein, DEFA5 could exert anti-tumor effects by inhibiting the cell mitosis. BMI1 serves as a transcription inhibitor of CDKN2A (30), which is a tumor suppressor gene and could arrest the cell cycle progression. Therefore, by antagonizing BMI1 expression, DEFA5 could indirectly call a halt to the aberrant cell division. In an experiment of gastric cancer cells, DEFA5 overexpression dramatically increased the number of G1-phase cells but significantly decreased G2/M-phase cells (28), indicating that DEFA5 overexpression could result in cell cycle arrest at the G1 phase. Given the role of DEFA5 in cell cycle arrest, it may be a novel target for developing new drugs, but further exploration is needed to clarify its effect in LUAD.

The tumor microenvironment, composed of tumor cells, immune cells, and stromal cells, is closely correlated with the response to immunotherapies and drug resistance (31). We found that the wild group had a higher proportion of monocytes and DCs through immune cell infiltration analysis. As the most important antigen-presenting cells in our body, DCs play a significant role in both innate and adaptive immunity. By presenting the antigen-MHC complex to the T cells, DCs could activate the T cell-mediated immune response to kill cancer cells (32). What’s more, by secreting high levels of type-I interferons and chemokines such as CXCL9 and CXCL10, DCs could facilitate the recruitment of effector T cells and NK cells into tumors and maintain the cytotoxic functions of effector cells (33, 34). Sipuleucel-T, a DC-based vaccine, has shown clinical benefits and been approved by the US Food and Drug Administration (FDA) for treating prostate cancer (27). As the stunning anti-tumor effects DC could elicit, it can be employed for killing cancer cells and may greatly improve the prognosis of LUAD patients.

Notably, even though the mutation group had a higher level of mutational load, fewer immune cells were detected than the wild group. Emerging evidence has demonstrated the effect of RB1 on immunity. The accelerated apoptosis of the immature T cells and a considerable drop in T cell number were observed in RB1-deficient zebrafish, which could be reversed by E2F1 knockdown (35). A single-institution investigation of NSCLC patients revealed the negative correlation between RB1 mutation and response to immunotherapy (36). Similarly, RB depletion in cancer cells impairs the immune response to a variety of stimuli in hepatocellular carcinoma (37). What’s more, the less immune cell infiltrated state in the mutation group could also be partially attributed to the higher level of immune checkpoints and lower level of co-stimulators.

Since sustaining proliferative signals is a hallmark of cancer (7), inhibition of the dysregulated cell division is a promising strategy for cancer therapies. The administration of CDK4/6 inhibitors could prevent the phosphorylation of RB1 by CDK-cyclin complexes, calling a halt to the cell cycle progression. Up to date, three generations of CDK inhibitors have been developed for cancer treatment. As the third-generation selective CDK inhibitors, Palbociclib, ribociclib, and abemaciclib have been approved by the US FDA to treat breast cancer (38). Despite the clinical benefit in specific breast cancer patients, the results of clinical trials accessing the effect of single-agent CDK inhibitors in NSCLC are frustrating (39). To fully evaluate the application of CDK inhibitors in NSCLC, further exploration in this field may target combination with other anti-cancer therapies and the discovery of predictive biomarkers.

There are also some limitations of our study. Besides CDKN2A, CCND1, CDK4, CCNE1, and RB1, other cell-cycle-related variants could also contribute to differences between the two groups, but they were neglected. Besides, patients in the mutation group were quite fewer than the wild group, so further investigation is needed in larger groups.

## Conclusion

In conclusion, we analyzed the differences in somatic mutations, genomic expression, and immune cell infiltration between the CDKN2A-CDK/cyclin-RB1 cell cycle progression pathway altered/non-altered patients with LUAD. Patients in the two groups have different somatic mutation and gene expression patterns, profiling of immune cell infiltration, and biological pathways that may play an important role in oncogenesis and tumor metastasis. We hope our study could improve our understanding of the function of the cell cycle progression pathway, thus contributing to the development of new therapies and precision medicine in the future.

## Data Availability Statement

The datasets presented in this study can be found in online repositories. The names of the repository/repositories and accession number(s) can be found in the article/**Supplementary Material**.

## Ethics Statement 

The studies involving human participants were reviewed and approved by the ethical committees of Zhongshan Hospital. The patients/participants provided their written informed consent to participate in this study.

## Author Contributions

Conception and design were contributed by CZ, YZ, and HF. Administrative support was contributed by YZ and HF. Provision of patients was contributed by HZ and DZ. Collection and assembly of data were contributed by GS and GB. Data analysis and interpretation were contributed by GS, GB, YB, BV, and GY. Draft and final approval of the manuscript were contributed by all authors.

## Funding

This research was supported by Xiamen Science and Technology Plan Guidance Project, Xiamen Municipal Science and Technology Commission under Grant [No. KZXD201922].

## Conflict of Interest

The authors declare that the research was conducted in the absence of any commercial or financial relationships that could be construed as a potential conflict of interest.

## Publisher’s Note

All claims expressed in this article are solely those of the authors and do not necessarily represent those of their affiliated organizations, or those of the publisher, the editors and the reviewers. Any product that may be evaluated in this article, or claim that may be made by its manufacturer, is not guaranteed or endorsed by the publisher.

## Supplementary Material

Supplementary Figure 1 Summary of the LUAD patients’ somatic mutation data, including the number of variants in each sample and variant types. **(A)** The mutation group. **(B)** The wild group.

Supplementary Figure 2 Volcano map of differentially expressed miRNAs **(A)** and differentially expressed lncRNAs **(B)**.

Supplementary Figure 3 Competing endogenous RNA (ceRNA) network of DEGs - differentially expressed miRNAs - differentially expressed lncRNAs.

Supplementary Figure 4 Boxplot of 22 immune cells between the mutation and wild groups from CIBERSORT. **P<0.05; **P<0.01; ***P<0.001.*

Supplementary Figure 5 Construction of the predictive model. **(A)** Coefficient profiles of variables in the LASSO regression model. **(B)** Tenfold cross-validation for turning parameter (λ) selection in the LASSO regression model. The partial likelihood deviance is plotted in log(λ), in which vertical lines are shown at the optimal values by minimum criteria and 1−SE criteria. **(C)** Nomogram of the predictive model. **(D)** Receiver operating characteristic curve (ROC) of the LASSO-binary logistic regression model.

Supplementary Figure 6 The expression levels of the 15 genes in the predictive model between the tumor and normal tissue generated by GEPIA webtools. **P<0.05.*

## Abbreviations

NSCLC, non-small cell lung cancer; LUAD, lung adenocarcinoma; TCGA, The Cancer Genome Atlas; DEG, Differentially expressed gene; CDK, cyclin-dependent kinase; CDKN2A, cyclin-dependent kinase inhibitor 2A; CCND1, cyclinD1; CDK4, cyclin-dependent kinase 4; CCNE1, cyclinE1; RB1, retinoblastoma 1; DMGs, differentially mutated genes; CNV, copy number variation; SNP, single nucleotide polymorphisms; Indels, insertions and deletions; GO, Gene Ontology; KEGG, Kyoto Encyclopedia of Genes and Genomes; GSEA, gene set enrichment analysis; GSVA, gene set variation analysis; PPI, Protein-to-protein interaction; MCODE, Molecular Complex Detection; ceRNA, competing endogenous RNA; lncRNA, long non-coding RNA; LM22, leucocyte signature matrix; ccDEGs, cell-cycle-related DEGs; LASSO, least absolute shrinkage and selection operator; ROC, receiver operating characteristic curve; DEPMAP, the Cancer Dependency Map; DCs, dendritic cells; AUC, area under the curve; FDA, Food and Drug Administration.
