# Supplementary figures and images for: Genomic And Tumor Microenvironment Differences Between Cell Cycle Progression Pathway Altered/Non-Altered Patients With Lung Adenocarcinoma
Source: Front Oncol. 2022 Feb 28;12:843528. doi: 10.3389/fonc.2022.843528 (PMC8919059; doi:10.3389/fonc.2022.843528)

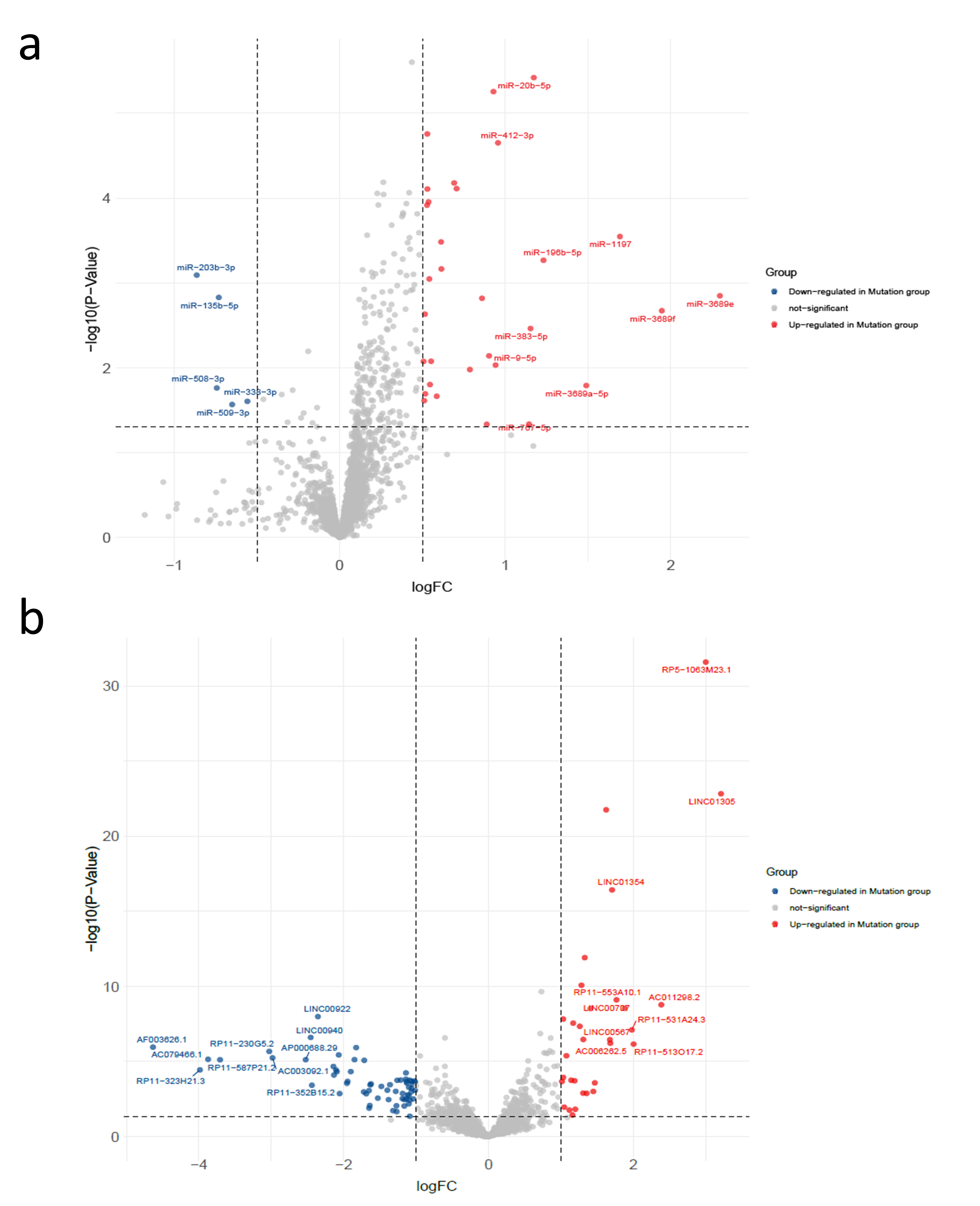

Supplement: Supplementary Figure 2 — Volcano map of differentially expressed miRNAs (A) and differentially expressed lncRNAs (B). [file Image_2.tif]

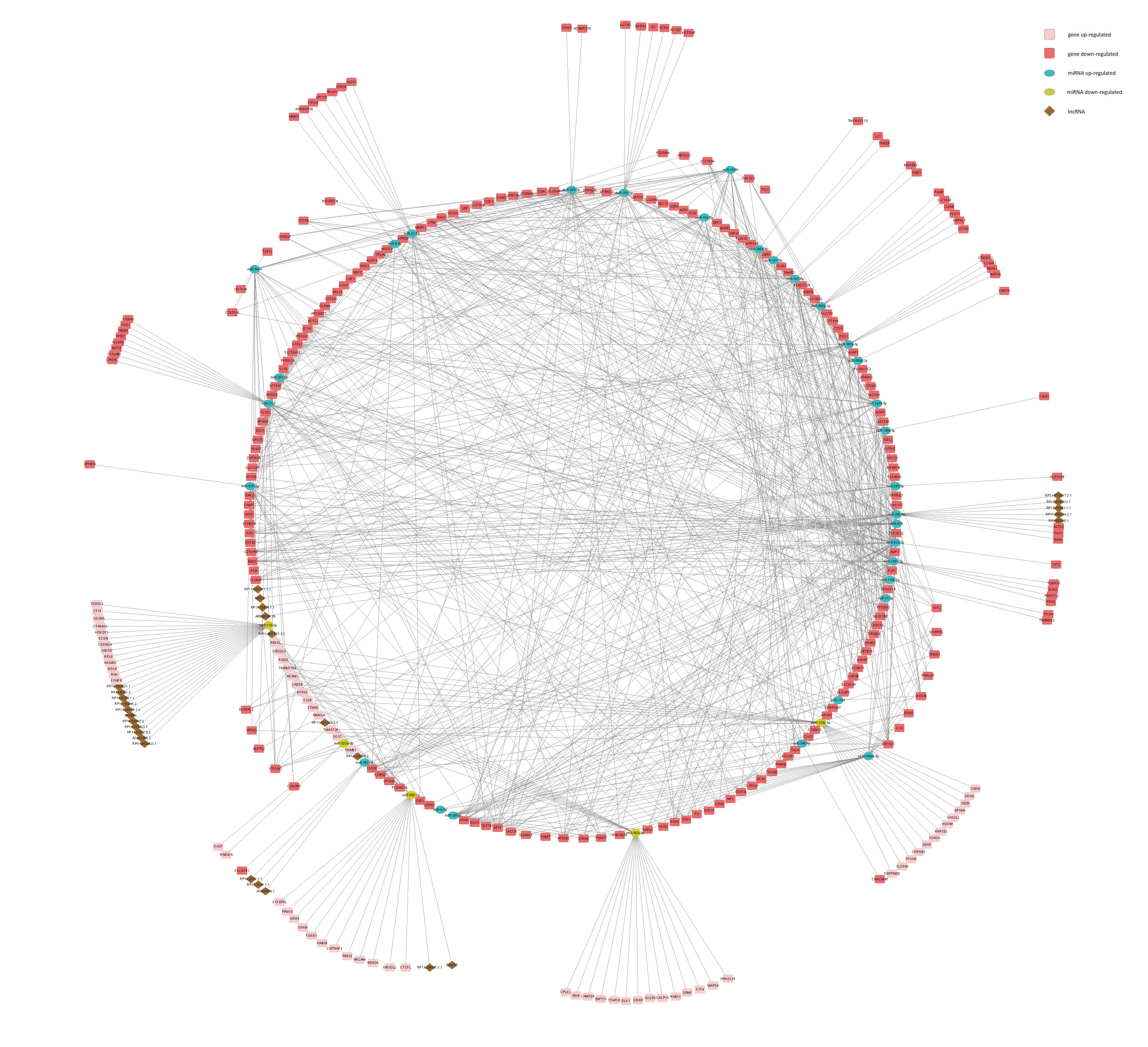

Supplement: Supplementary Figure 3 — Competing endogenous RNA (ceRNA) network of DEGs - differentially expressed miRNAs - differentially expressed lncRNAs. [file Image_3.tiff]

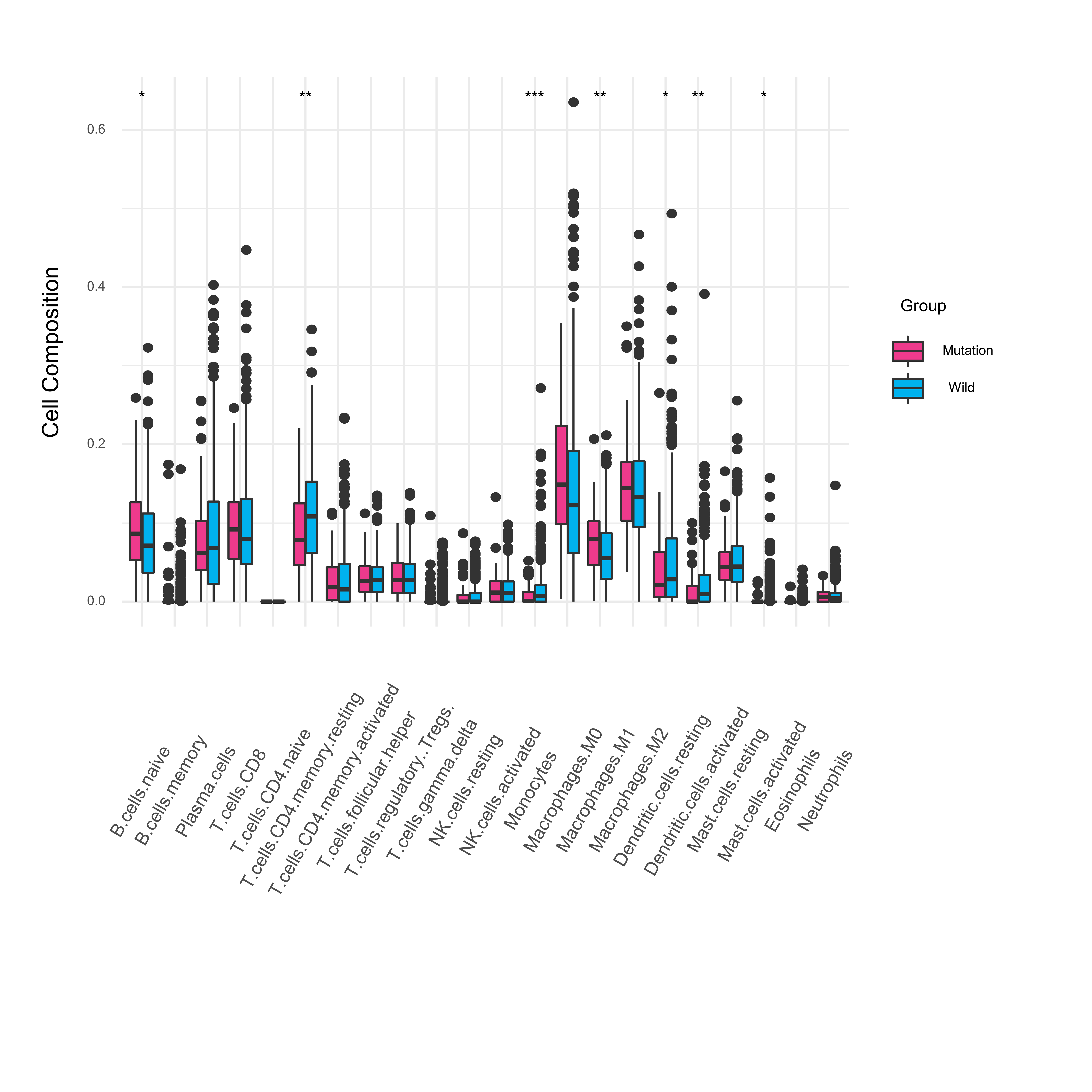

Supplement: Supplementary Figure 4 — Boxplot of 22 immune cells between the mutation and wild groups from CIBERSORT. *P<0.05; **P<0.01; ***P<0.001. [file Image_4.tif]
